# Preclinical investigation of anti-tumor efficacy of allogeneic natural killer cells combined with cetuximab for head and neck squamous cell carcinoma

**DOI:** 10.1007/s00262-025-03959-8

**Published:** 2025-03-10

**Authors:** Chaeyeon Kim, Mina Han, Gamin Kim, Wonrak Son, Jeongah Kim, Minchan Gil, Yong-Hee Rhee, Nam Suk Sim, Chang Gon Kim, Hye Ryun Kim

**Affiliations:** 1 Division of Medical Oncology, Department of Internal Medicine, Yonsei University College of Medicine, 50-1 Yonsei-ro, Seodaemun-gu, Seoul, 03722 Republic of Korea; 2 Department of Oncology, Yonsei University College of Medicine, Seoul, Republic of Korea; 3 NKMAX Co., Ltd., Seongnam, Republic of Korea; 4 Department of Otorhinolaryngology, Severance Hospital, Yonsei University College of Medicine, Seoul, Republic of Korea; 5 Division of Medical Oncology, Department of Internal Medicine, Yonsei Cancer Center, Yonsei University College of Medicine, Seoul, Republic of Korea

**Keywords:** Head and neck squamous cell carcinoma, Allogeneic natural killer cell, Cetuximab, Antibody-dependent cellular cytotoxicity

## Abstract

Head and neck squamous cell carcinoma (HNSCC) presents a significant therapeutic challenge because of the limited effectiveness of current treatments including immunotherapy and chemotherapy. This study investigated the potential of a novel combination therapy using allogeneic natural killer (NK) cells and cetuximab, an anti-epidermal growth factor receptor monoclonal antibody, to enhance anti-tumor efficacy in HNSCC. Allogeneic NK cells were tested against HNSCC cells in vitro and NOG (NOD/Shi-scid/IL-2Rγ null) xenograft mouse models for cytotoxicity. In vitro assays demonstrated enhanced cytotoxicity against HNSCC cells when NK cells were combined with cetuximab, a phenomenon attributed to antibody-dependent cellular cytotoxicity. In vivo, the combination therapy exhibited a significant anti-tumor effect compared to either monotherapy, with high NK cell infiltration and cytotoxic activity in the tumor microenvironment. Tumor infiltration by NK cells was confirmed using flow cytometry and immunohistochemistry, highlighting the increased presence of NK cells (CD3^−^ CD56^+^). These findings suggest that combination allogeneic NK cells and cetuximab could be a potential therapeutic modality for HNSCC and provide a foundation for future clinical trials to improve patient outcomes.

**Supplementary Information:**

The online version contains supplementary material available at 10.1007/s00262-025-03959-8.

## Introduction

Head and neck squamous cell carcinoma (HNSCC) is the seventh most common cancer worldwide [1]. Current systemic treatment strategies for HNSCC include cytotoxic-chemotherapy and/or immunotherapy [2]. Immunotherapy, specifically immune checkpoint inhibitors targeting the programmed death-1 and cytotoxic T-lymphocyte-associated protein 4 pathways [3], allows immune cells to effectively recognize and attack cancer cells. Although these therapies have demonstrated promising ability to improve the outcomes of patients with recurrent or metastatic disease, they are incompletely effective, with only 20–30% of patients responding to immunotherapy [4]; thus, an unmet demand remains. Cetuximab, an antibody targeting the epidermal growth factor receptor (EGFR), is also used to treat patients with head and neck cancer [5]. Despite a targeted approach, its effectiveness as monotherapy remains limited, highlighting the need for optimized combination strategies to improve patient outcomes.

Natural killer (NK) cells have garnered attention as a promising immune cell treatment because of their selective cytotoxicity against cancer cells. They play a key role in regulating inflammation and immune responses by producing cytokines including interferon-γ and tumor necrosis factor-α and can inhibit cancer cell proliferation and metastasis. Moreover, they eliminate cancer stem cells, which are crucial for recurrence [6]. Adoptive NK cell therapies involve both autologous and allogeneic approaches. Autologous NK cells often encounter challenges, including self-recognition of human leukocyte antigens (HLA), leading to primary and/or acquired resistance [7]. Allogeneic NK cells from donors with non-matching NK cell immunoglobulin-like receptor (KIR)–HLA combinations can bypass these issues, enhancing anti-tumor activity with a minimal risk of graft-versus-host disease [8–11]. Although autologous NK cell therapy has a low immune system rejection rate, cell isolation and expansion require time, which can affect treatment feasibility. Allogeneic NK cell therapy using cells from healthy donors may offer reduced cytotoxicity and improved efficacy [12, 13].

Cetuximab induces antibody-dependent cellular cytotoxicity (ADCC) [14–16], a mechanism primarily mediated by NK cells. In cancer patients, the detection of reduced NK cell activity [17] led to the hypothesis that the injection of allogeneic instead of autologous NK cells would enhance ADCC efficacy in cetuximab combination therapy. Here, we found that allogeneic NK cells combined with cetuximab demonstrated enhanced anti-tumor efficacy compared to monotherapy through the ADCC effect using in vitro and in vivo experiments. Our results suggest that combination therapy with allogeneic NK cells and cetuximab is promising for improving treatment outcomes in patients with HNSCC.

## Materials and methods

### NK cell expansion

SNK02, an allogeneic NK cell product, was manufactured under good manufacturing practice conditions (NKMAX, Seongnam, Korea) for in vivo animal testing, following previously described methods with certain modifications [18]. Briefly, CD3^−^/CD56^+^ cells were isolated from the peripheral blood mononuclear cells (PBMCs) of the enrolled healthy donors using CliniMACS microbeads (Miltenyi Biotech GmbH) following the manufacturer’s instructions and cultured in Roswell Park Memorial Institute (RPMI)-1640 medium (WELGENE Inc.), supplemented with 10% fetal bovine serum (FBS) (Hyclone), 20 μg/mL gentamicin (Gibco), γ-irradiated (100 Gy)—KL-1 and LCL feeder cells, 500 IU/mL interleukin (IL)-2 (PROLEUKIN, Novartis, Basel, Switzerland), and 50 ng/mL IL-21 (NKMAX Co.). NK cells were subcultured every 3–4 days in fresh RPMI-1640 medium supplemented with IL-2. After 14 days of culture, the cells were harvested and either re-expanded with feeder cells in the presence of cytokines for another 14 days or cryopreserved for further expansion following the treatment schedule. On day 28 of total culture, the cells were harvested again and cultured with two types of feeder cells and cytokines for an additional 17–18 days. The expanded NK cells were collected on day 45 or 46 of the expansion culture, excluding any cryopreservation period. They were then washed three times, resuspended in a cryopreservation medium containing human serum albumin (Green Cross Corp.) and CryoStor® cell cryopreservation media (Sigma), transferred to cryopreservation bags, and cryopreserved using an automatic freezing system (CRF). Once thawed, the cells were then used for further experiments.

### Cell culture

FaDu/Luciferase (RRID:CVCL_1218; NKMAX Co.) were cultured in RPMI-1640 medium (Corning), supplemented with 10% heat-inactivated FBS (Gibco) and 1% penicillin–streptomycin (Gibco). The cells were cultured at 37 °C and 5% carbon dioxide (CO_2_). The FaDu cells were subcultured twice per week. The culture medium was removed, and the cells were gently washed with warm phosphate-buffered saline (PBS) (WELGENE Inc.). The cells were detached from the dish using 0.25% trypsin-ethylenediaminetetraacetic acid (Gibco) and subsequently neutralized in medium. The cells were centrifuged, pelleted, resuspended in fresh medium, and cultured in a 100-mm dish. All experiments were performed with mycoplasma-free cells.

### Cytotoxicity assay

FaDu/Luciferase cells were stained with 10 μM calcein-AM (C1430) and were seeded (5 × 10^3^) in 96-well clear flat bottom plates (Corning) and incubated overnight at 37 °C and 5% CO_2_. FaDu/Luciferase cells were treated with cetuximab and co-cultured with NK cells at various effector-to-target ratios (1:1, 3:1, and 10:1). After 4 h, the supernatant was transferred to a 96-well black/clear-bottom plate. The fluorescence intensities at 488 and 594 nm were measured using a microplate reader (Varioskan LUX). NK cell-mediated killing of FaDu/Luciferase cells was observed in real time using a thin imager (Leica).

### In vivo* experiment*

Six-week-old female NOG mice were purchased from the Central Institute for Experimental Animals (CIEA, Japan). All animal experiments were approved by the local Institutional Animal Care and Use Committee. To generate tumors, FaDu/Luciferase cells (2 × 10^6^ cells/100 μL) were injected subcutaneous (s.c.) into the flank of each mouse. Tumor size was measured three times per week using electronic digital calipers. The mice were injected intraperitoneally with PBS and 2.5 mg/kg cetuximab and s.c. into the flank with IL-2 (8.4 × 10^4^ IU/mouse). The classification of high and low doses of NK cells was based on a previous clinical study evaluating SNK01 in combination with pembrolizumab for non-small cell lung cancer [19]. Using the clinical dose from this study as a reference, we calculated the equivalent dose for mice based on body weight conversion. In this model, the low dose was determined to be 1.3 × 10⁶ cells (~ 1 × 10⁶ cells), and the high dose was defined as 10 times the low dose, or 1 × 10⁷ cells. All in vivo experiments were performed at least twice.

### Calculating percentage change in the tumor growth inhibition

To calculate tumor growth inhibition, the percentage change from mean tumor size of control group was calculated using tumor measurements taken at 24 or 31 days post-tumor injection. In this formula, “0%” represents the mean tumor size of control group at the specified time point that serves as the reference value against which treatment responses are assessed. The formula used to calculate the percentage change in the tumor growth inhibition was as follows:

$$=-(100-\left(\frac{\text{tumor size at day }24\text{ or }31}{\text{mean tumor size of control group}}\right)\times 100)$$

### IVIS spectrum imaging

Bioluminescence imaging was performed weekly using the IVIS Spectrum (PerkinElmer, USA). The mice were intraperitoneally injected with d-luciferin (150 mg/kg). The bioluminescence imaging was performed on mice anesthetized with 2% isoflurane. Total counts were measured using Living Image software (PerkinElmer).

### Immunohistochemistry staining

Mouse tumor tissues were fixed overnight in 4% formaldehyde. Tissue sections were stained with Ki-67anti-human antibody (9027S) purchased from Cell Signaling Technology. Human NKp46/NCR1 (MAB1850-100) was purchased from R&D Systems. Immunohistochemistry (IHC) images were obtained using a BX43 light microscope (Olympus, Tokyo, Japan), and Ki-67-positive cells were objectively counted.

### Flow cytometry analysis

The flow cytometry was performed using a CytoFLEX LX flow cytometer (Beckman Coulter). The data were analyzed using FlowJo software. The cells were washed with cold flow cytometry buffer (PBS supplemented with 2% FBS) and stained with fluorochrome-conjugated anti-human CD56 (740,171), CD16 (561,304), CD45 (557,748), and CD3 (561,807) antibodies purchased from BD Biosciences. EGFR (352,928) was purchased from BioLegend, whereas the LIVE/DEAD™ Fixable Near-IR Dead Cell Stain Kit was purchased from Invitrogen. The antibodies were diluted 1:50 and incubated for 20 min at 4 °C in the dark.

### Statistics

For the survival analysis, the log-rank (Mantel–Cox) test was used to compare differences. Data are presented as the mean ± standard deviation or standard error of the means of the experimental replicates. Student’s t test was used to compare the two groups. Two-way analysis of variance (ANOVA) was used to compare three or more independent groups using GraphPad Prism v8 software (GraphPad, San Diego). Statistical significance was set at *p* < 0.05.

## Results

### Allogeneic NK-high cells effectively managed HNSCC in NOG xenograft mouse model

**Fig. 1:**
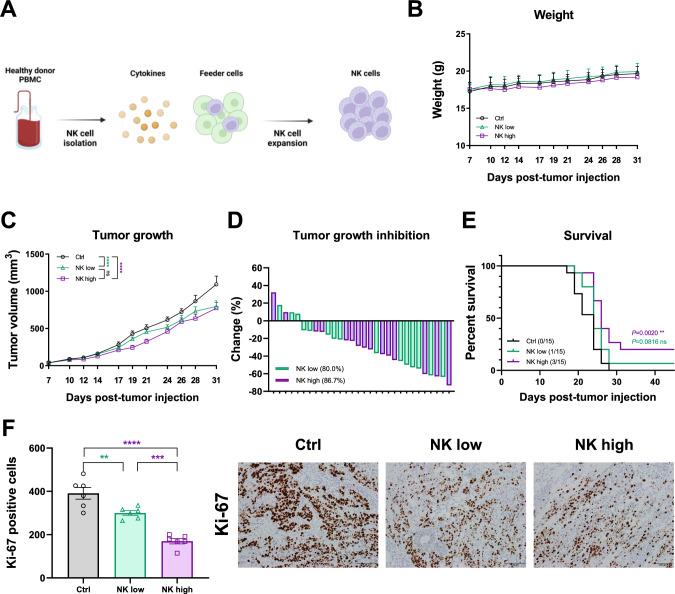
High-dose allogeneic NK cells effectively controlled head and neck tumors in a NOG xenograft mouse model. **A** Scheme of NK isolation and expansion. **B** Graphs of mouse weights. **C** Graphs of mouse tumor growth. Statistical significance was determined by two-way analysis of variance (ANOVA). **D** Graphs of tumor growth inhibition. **E** Graphs of survival rate. *N* = 15 mice per group. Statistical significance was determined by two-way ANOVA or a paired t test. **F** Immunohistochemistry staining with Ki-67 antibody of the mice tumor and summary graph (×20)

In the current study, we first assessed the feasibility of allogeneic NK cell transplantation. NK cells were isolated from PBMCs from healthy donors and expanded using cytokines and feeder cells. This expansion process significantly increased the number of NK cells (3.16 ± 0.67 × 10^10^ folds), ensuring sufficient quantities for subsequent experiments (Fig. 1A).

Next, we evaluated the ability of various allogeneic NK cell doses to control HNSCC in a NOG xenograft mouse model. The study included control (Ctrl), low-dose NK (NK-low; 1 × 10^6^ cells/mouse), and high-dose NK (NK-high; 1 × 10^7^ cells/mouse) groups. The body weights of the mice were evaluated throughout the experiment to monitor systemic toxicity. All groups maintained stable body weights without significant differences among them (Fig. 1B), indicating that NK cell treatment did not cause notable adverse effects, even at high doses. Tumor growth was significantly reduced in the NK-low and NK-high groups versus the Ctrl group (Fig. 1C, S1A).

Analysis of the tumor growth inhibition revealed a notable difference. The NK-high group exhibited a higher tumor growth inhibition (86.7%) than that noted in the NK-low group (80.0%), highlighting the enhanced efficacy of the elevated dose NK cell treatment (Fig. 1D). Although the NK-low group exhibited no meaningful improvement in survival versus the Ctrl group, the NK-high group had a clear survival advantage (Fig. 1E), highlighting the efficacy of the increased dose of NK cells.

We also evaluated the effect of NK cells on tumor cell proliferation. Ki-67 staining revealed the highest inhibitory effect on tumor cell proliferation in the NK-high group, with fewer Ki-67-positive cells than in the NK-low group (Fig. 1F). Based on these findings, an increased dose of NK cells was selected for subsequent studies owing to its superior ability to improve survival outcomes and inhibit tumor cell proliferation despite no marked differences growth between the NK-low and NK-high groups.

### NK-high and IL-2 combination therapy did not significantly enhance anti-tumor response

**Fig. 2:**
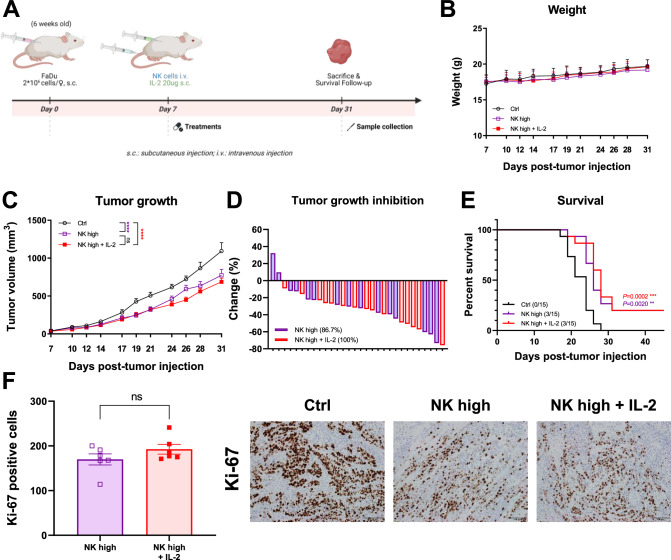
Combination therapy consisting of high-dose NK cells and IL-2 did not enhance the anti-tumor response. **A** Study design for combination therapy of allogeneic NK cells and IL-2. **B** Graphs of mouse weight. **C** Graphs of mouse tumor growth. Statistical significance was determined by two-way ANOVA. **D** Graphs of tumor growth inhibition. **E** Graphs of survival rate. *N* = 15 mice per group. Statistical significance was determined by two-way ANOVA or a paired t test. **F** Immunohistochemistry staining with Ki-67 antibody of the mice tumor and summary graph (×20)

Following the selection of an increased dose of NK cells based on our earlier findings, we investigated IL-2 as a partner drug to enhance NK cell activity in vivo (Fig. 2A). As IL-2 is widely utilized to expand allogeneic NK cells, we hypothesized that combining it with NK-high infusions would enhance the anti-tumor response. The weights of the mice were monitored to evaluate their overall health and the potential treatment-related side effects (Fig. 2B). However, a tumor growth analysis revealed no significant (Fig. 2C, S1A).

Not only that, no meaningful reduction in tumor growth inhibition or improvement in survival was observed in the combination therapy versus NK-high group (Fig. 2D, 2E).

Furthermore, IHC staining using the Ki-67 antibody to assess tumor cell proliferation demonstrated no significant differences between the combination therapy and NK-high groups (Fig. 2F). Similar experiments were conducted using reduced doses of NK cells combined with IL-2, and the results mirrored those of the NK-high group. (Fig. S2A–E).

These results collectively indicate that IL-2, when combined with NK-high or NK-low, did not augment the anti-tumor response in vivo. The lack of an improved anti-tumor response through the IL-2 combination in vivo prompted us to investigate other combination treatment partners.

### *Cetuximab contributes to ADCC in NK cell-mediated cytotoxicity *in vitro

**Fig. 3:**
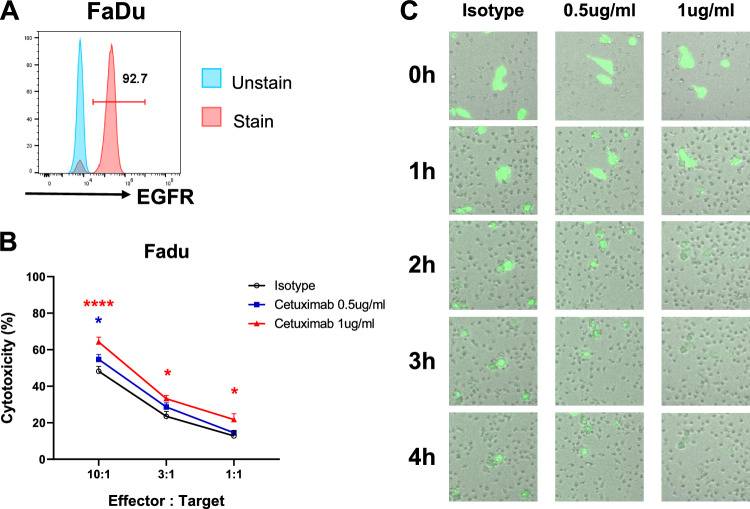
Cetuximab enhanced cytotoxic effect of allogeneic NK cells in vitro. **A** Expression of epidermal growth factor receptor on FaDu cells. **B** Antibody-dependent cellular cytotoxicity assay using calcein-AM for 4 h at 10:1, 3:1, and 1:1 effector-to-target ratios of allogeneic NK cells:FaDu cells. **C** Images of FaDu cells (stained green by calcein-AM) being co-cultured with NK cells

Cetuximab, an EGFR-targeting antibody, was selected for subsequent experiments due to its potential to enhance NK cell-mediated ADCC. In preparation for the subsequent experiments with cetuximab, we first confirmed the EGFR expression in FaDu cells. The expression of EGFR in FaDu cells (Fig. 3A) provided a suitable rationale for using cetuximab as the drug-induced ADCC when combined with NK cells. In vitro experiments confirmed the ADCC effect, as confirmed by the results of the calcein-AM assay, where NK cells demonstrated effective cytotoxicity against FaDu cells at effector-to-target (E:T) ratios (10:1, 3:1, and 1:1) (Fig. 3B). Images of FaDu cells co-cultured with NK cells further illustrated this effect (Fig. 3C).

Collectively, while IL-2 showed limitations in enhancing NK cell activity in vivo, the ADCC effect of cetuximab was enhanced by NK cell combination treatment in HNSCC.

### Synergistic effect of allogeneic NK cells and cetuximab on HNSCC suppression in NOG xenograft mouse model

**Fig. 4:**
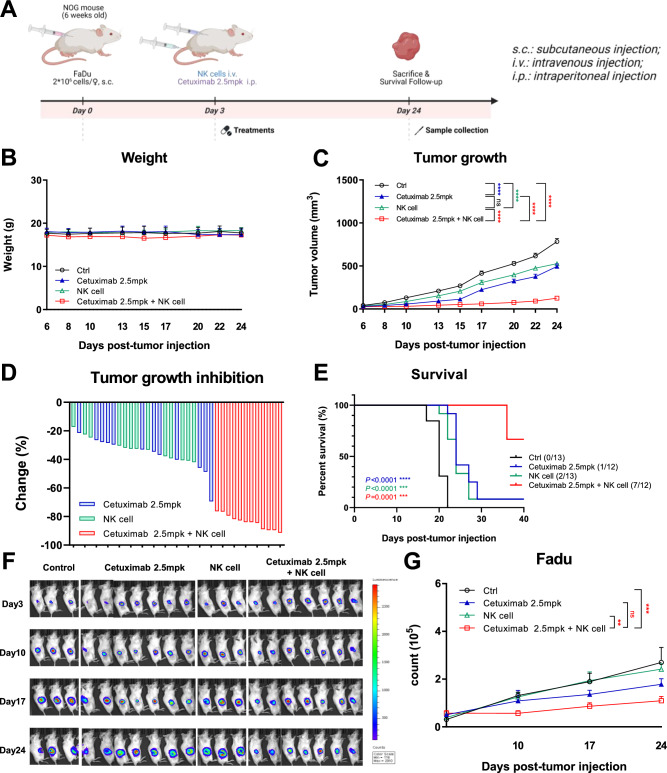
Combination therapy consisting of allogeneic NK cells and cetuximab effectively managed head and neck tumors in the NOG xenograft mouse model. **A** Study design for combination therapy of allogeneic NK cells and cetuximab. **B** Graphs of mouse weight. **C** Graphs of mouse tumor growth. Statistical significance was determined by two-way ANOVA. **D** Graphs of tumor growth inhibition. **E** Graphs of survival rate. *N* = 12–13 mice per group. Statistical significance was determined by two-way ANOVA or a paired t test. **F** IVIS spectrum images. **G** Graphs of tumor burden (count). *N* = 12–13 mice per group

Building on previous experiments in which NK-high cells were selected, we investigated the combination of allogeneic NK cells and cetuximab in the NOG xenograft mouse models to evaluate the ADCC effect and overall therapeutic efficacy of the combination treatment (Fig. 4A). The study included four groups: ctrl, cetuximab 2.5 mg/kg (mpk), NK cells (1 × 10^7^ cells/mouse), and cetuximab 2.5mpk + NK cell.

The weights of the mice were monitored to evaluate their overall health and potential treatment-related side effects (Fig. 4B). No significant weight loss was observed in any groups, suggesting that the treatment was well tolerated. Additionally, the cetuximab + NK cell group exhibited substantial reduction tumor growth compared with that in the control and single-agent groups (cetuximab and NK cells alone) (Fig. 4C, S3A). The tumor growth inhibition was notably greater in the cetuximab + NK cell group than in the other groups (Fig. 4D), and survival analysis revealed improved survival in the cetuximab + NK cell group than in the control and monotherapy groups (Fig. 4E). This demonstrated that the therapeutic benefit was statistically significant and highlighted the superior effect of combination therapy. The reduced tumor burden was visually identified in the cetuximab + NK cell group (Fig. 4F), and the results were further confirmed by quantitative analysis using IVIS spectrum imaging (Fig. 4G).

Collectively, the combination of allogeneic NK cells and cetuximab effectively managed HNSCC in NOG xenograft mouse model, showing significant reductions in tumor growth and improved survival rates. These results highlight the potential of this combination therapy to enhance anti-tumor efficacy.

### Enhanced NK cell infiltration and ADCC in HNSCC NOG xenograft mouse model treated with allogeneic NK cells and cetuximab

**Fig. 5:**
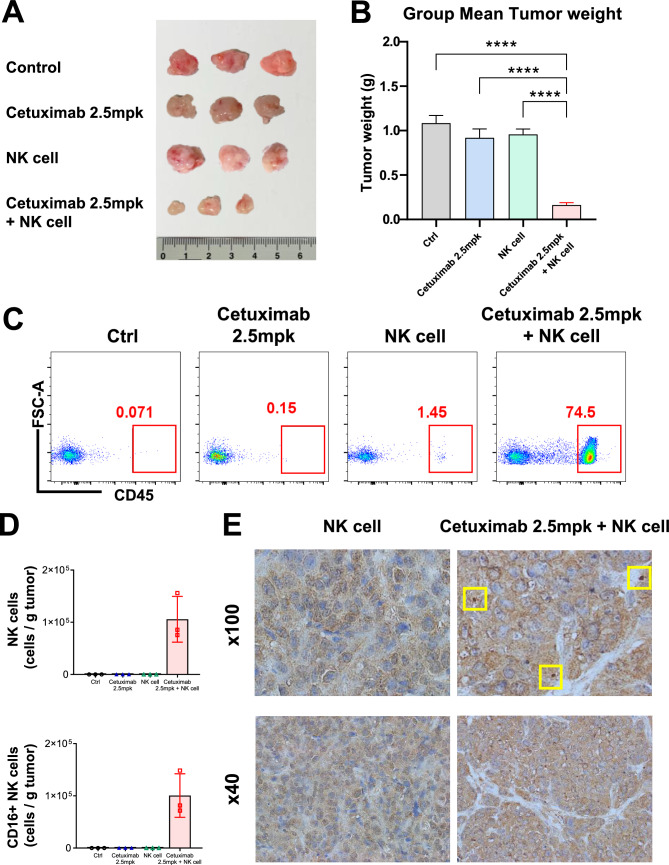
Allogeneic NK cells infiltrated tumors via antibody-dependent cellular cytotoxicity. **A** Tumors in each group of mice. **B** Tumor weights. **C** Flow cytometry analysis revealed the proportion of CD45^+^ cells in the tumors. **D** Graphs of flow cytometry results displaying the proportion of NK (CD3^−^ CD56^+^) and cytotoxic NK (CD16^+^) cells within the tumors. **E** Immunohistochemistry staining with NKp46 antibody of the mice tumor (×40, ×100)

Following the initial experiments demonstrating the therapeutic efficacy of allogeneic NK cells combined with cetuximab, we conducted further investigations to assess NK cell infiltration and ADCC within tumors (Fig. 5A). Differences in tumor size all groups were visually identified, and the cetuximab + NK cell group had a markedly lower mean tumor weight than the other groups (Fig. 5B). This tumor weight reduction is consistent with previous findings. Flow cytometry analyzed the composition of immune cells within the tumor. The proportion of CD45^+^ (marker for human immune cell) cells was assessed, and immune cell penetration was augmented in the cetuximab + NK cell group compared to that in the other treatment groups (Fig. 5C). Further analysis focused on NK cell infiltration, and the proportions of NK (CD3^−^ CD56^+^) and cytotoxic NK (CD3^−^ CD56^+^ CD16^+^) cells were significantly increased in the cetuximab group with NK cells (Fig. 5D, S4A, S4B). This increase in the number of NK cells within the tumor microenvironment supports the hypothesis that combined treatment enhances NK cell-mediated cytotoxicity via ADCC.

The IHC analysis of the tumor sections confirmed the presence of NK cells within the tumor by staining with NKp46 antibody. Compared to the NK cell monotherapy group, the addition of cetuximab enhanced NK cell infiltration within the tumor (Fig. 5E). In summary, the combination of allogeneic NK cells and cetuximab reduced tumor size and prolonged survival and enhanced NK cell infiltration and ADCC within the tumor microenvironment, highlighting the potential of this combination therapy to treat HNSCC.

## Discussion

This study used a NOG xenograft mouse model to demonstrate the enhanced anti-tumor efficacy of combined allogeneic NK cell and cetuximab treatment for HNSCC. During the selection process for allogeneic NK cells and their partner drugs, IL-2 (commonly used for NK cell expansion) was selected and tested in the xenograft mouse model. However, IL-2 alone is insufficient to significantly enhance NK cell anti-tumor activity, potentially due to its role in promoting regulatory T cell (Treg) expansion through IL-2Rα, which is highly expressed in Tregs. IL-2Rα activation by IL-2 may contribute to an immunosuppressive environment, limiting the efficacy of IL-2 on NK cells [20–22]. Moreover, *IL-2,* encoding IL-2, expression was associated with *FoxP3* in The Cancer Genome Atlas HNSCC cohort (Fig. S5A), which not only diminishes its immune activation effects but also increases suppressive populations, thereby reducing its overall efficacy. Recent approaches explored bypassing IL-2Rα activation by targeting the IL-2Rβ and IL-2Rγ subunits, highlighting the limitations of IL-2 [21]. This underscores the need for alternative combination strategies to overcome such immunosuppressive mechanisms. Therefore, the effects of cetuximab combined with NK cells were explored. The combination therapy resulted in significant tumor suppression and facilitated NK cell infiltration into tumors via ADCC.

Several approaches have been explored in the context of NK cell therapy for cancer treatment. Traditionally, autologous NK cells have been used; however, such approaches have significant limitations, particularly regarding cellular persistence and activity. Therapies aimed at enhancing NK cell activity, such as those involving the use of monalizumab, an immune checkpoint inhibitor targeting NKG2A [23–25], were recently introduced and subjected to clinical trials. Despite efforts to block NK cell inhibition signals, clinical trials of combination cetuximab treatment have not demonstrated appreciable efficacy compared to cetuximab monotherapy [26]. Given the limitations of autologous NK cells and therapies focusing solely on inhibitory checkpoint molecules, here we explored the preclinical activity of allogeneic NK cells.

Our findings highlight the advantages of using high-dose allogeneic NK cells combined with cetuximab. The current in vitro experiments demonstrated that the combination of cetuximab and NK cells significantly enhanced the cytotoxic effects against FaDu cells, a human HNSCC cell line. This synergy is likely attributable to the ability of cetuximab to bind to the EGFR on tumor cells, flagging them for destruction by NK cells.

In this study, we used NOG mice, which lack mature T, B, and NK cells, making them ideal models for human cell transplantation and cancer research. First, these in vivo experiments revealed that high doses of allogeneic NK cells were more effective at tumor suppression than lower doses. Therefore, high-dose allogeneic NK cells were used in subsequent experiments. Next, we investigated the effect of cetuximab and allogeneic NK-high cells on the tumor burden of NOG xenograft mice. Tumor growth was significantly suppressed in the combination versus Ctrl and monotherapy groups, providing additional evidence of the enhanced anti-tumor efficacy of this therapeutic strategy. Cetuximab not only inhibits tumor growth by blocking EGFR signaling, but also activates NK cells to induce ADCC [16]. This dual mechanism underscores the potential of combining cetuximab with NK cell-based immunotherapies to enhance anti-tumor efficacy. Previous studies demonstrated the synergistic effects of cetuximab and NK cells, showing that cetuximab enhances NK cell-mediated cytotoxicity, leading to improved anti-tumor responses across various cancer models [27–29]. Results were consistent with these findings, as the combination of allogeneic NK cells and cetuximab similarly induced significant tumor growth inhibition and improved survival in HNSCC models.

NK cell populations generally have characteristics such as CD56^+^ and CD16^+^, which are markers of cytotoxic potential; NKp46 is also being emphasized as a key marker of natural killer cytotoxicity [30, 31]. The proportions of CD45^+^ immune cells, NK cells (CD3^–^CD56^+^), and cytotoxic NK cells (CD16^+^) were significantly higher in the combination versus other treatment groups. These findings indicate that the combined therapy not only enhances NK cell infiltration, but also promotes cytotoxic activity within the tumor microenvironment. The IHC analysis provided additional evidence of enhanced NK cell activity and tumor suppression.

Cetuximab effectively induces ADCC and contributes to tumor suppression; however, its limitations as a therapeutic agent cannot be overlooked. Although cetuximab is known to cause skin toxicity in humans [5, 32], this adverse effect was not observed in these experimental mice, highlighting a limitation of preclinical models in fully replicating human side effects. These findings emphasized the importance of future clinical trials to monitor whether combination therapies, such as the one proposed in this study, could mitigate or exacerbate cetuximab-related toxicities.

In conclusion, this study provides compelling evidence that the combination of high-dose allogeneic NK cells and cetuximab significantly improves tumor suppression and enhances NK cell-mediated cytotoxicity via ADCC. Allogeneic NK cells, with their practical advantages over autologous cells, offer a promising therapeutic strategy for meeting the unmet needs of patients with HNSCC, especially those demonstrating a limited response to current treatments.

## Supplementary Information

Below is the link to the electronic supplementary material.

Supplementary file1 (PDF 13 KB)

Supplementary file2 (PDF 170 KB)

Supplementary file3 (PDF 9 KB)

Supplementary file4 (PDF 56 KB)

Supplementary file5 (PDF 75 KB)

Supplementary file6 (ZIP 453349 KB)

## Data Availability

The datasets generated during and/or analyzed during the current study are available from the corresponding author on reasonable request.
