# Supplementary figures and images for: Preclinical investigation of anti-tumor efficacy of allogeneic natural killer cells combined with cetuximab for head and neck squamous cell carcinoma
Source: Cancer Immunol Immunother. 2025 Mar 10;74(4):144. doi: 10.1007/s00262-025-03959-8 (PMC11893940; doi:10.1007/s00262-025-03959-8)

**A**

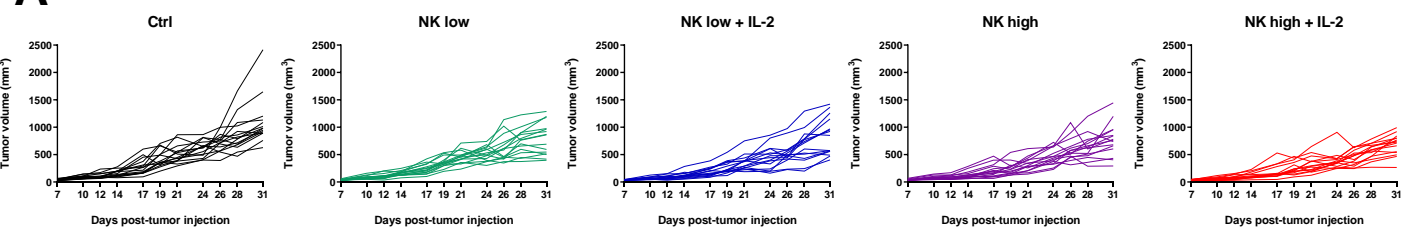

Supplement: Supplementary file 1 — Supplementary file1 (PDF 13 KB) [file 262_2025_3959_MOESM1_ESM.pdf]

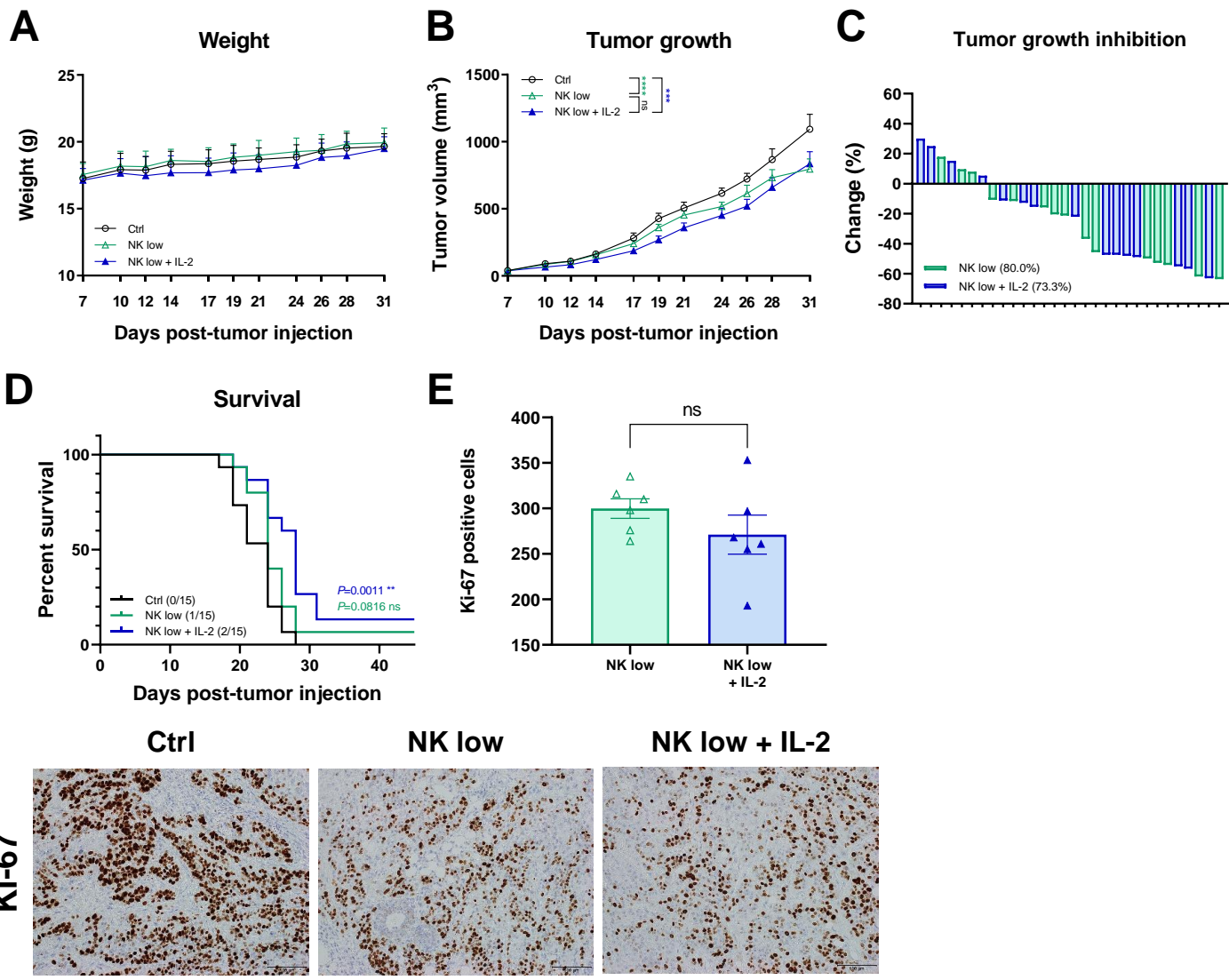

Supplement: Supplementary file 2 — Supplementary file2 (PDF 170 KB) [file 262_2025_3959_MOESM2_ESM.pdf]

A

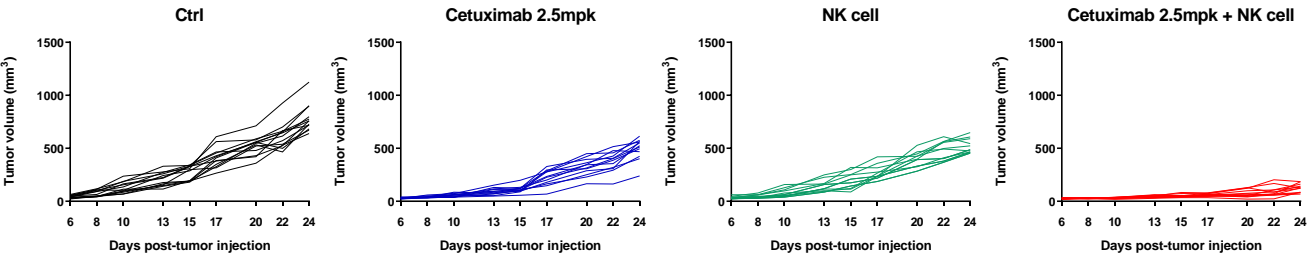

Supplement: Supplementary file 3 — Supplementary file3 (PDF 9 KB) [file 262_2025_3959_MOESM3_ESM.pdf]

**A**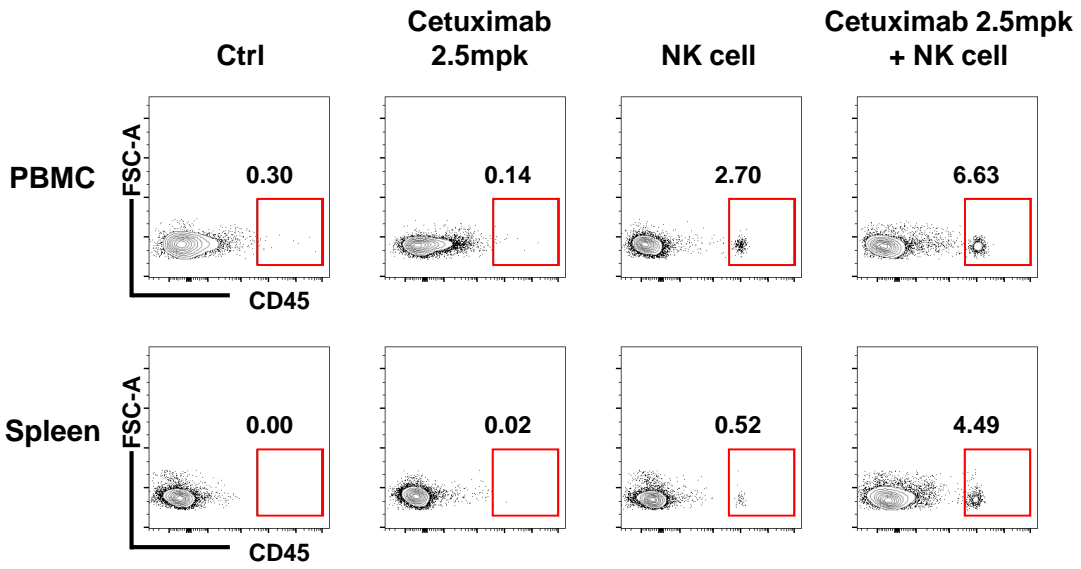**B**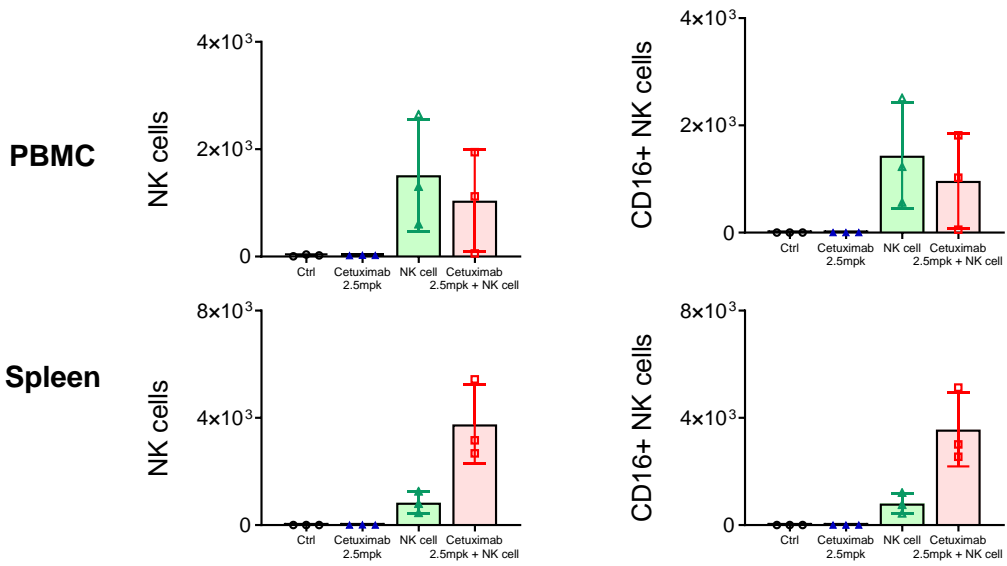

Supplement: Supplementary file 4 — Supplementary file4 (PDF 56 KB) [file 262_2025_3959_MOESM4_ESM.pdf]

**A**

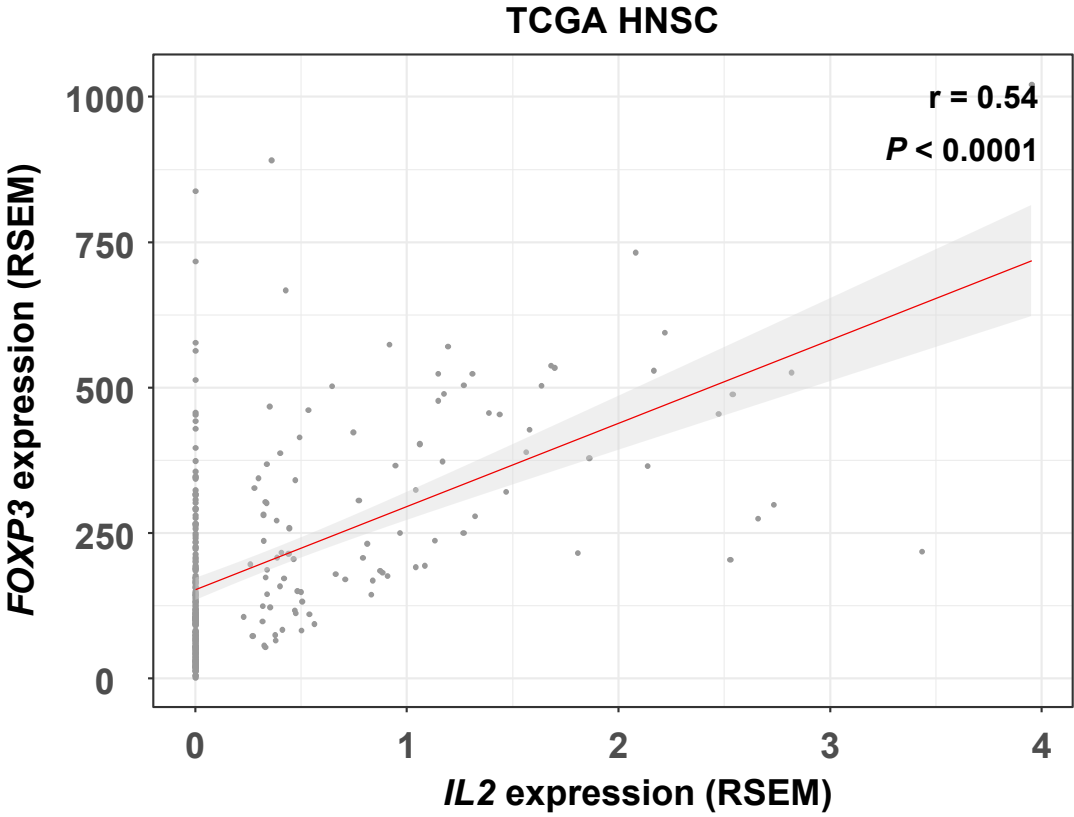

Supplement: Supplementary file 5 — Supplementary file5 (PDF 75 KB) [file 262_2025_3959_MOESM5_ESM.pdf]
