# Supplementary material for: Preclinical investigation of anti-tumor efficacy of allogeneic natural killer cells combined with cetuximab for head and neck squamous cell carcinoma
Source: Cancer Immunol Immunother. 2025 Mar 10;74(4):144. doi: 10.1007/s00262-025-03959-8 (PMC11893940; doi:10.1007/s00262-025-03959-8)
Supplement: Supplementary file 6 — Supplementary file6 (ZIP 453349 KB) [file 262_2025_3959_MOESM6_ESM.zip › oncoimmunology_movie_captions.pdf]

Movie S1.

Movie of FaDu cells (stained green by calcein AM) being co-cultured with NK cells; Isotype

Movie S2.

Movie of FaDu cells (stained green by calcein AM) being co-cultured with NK cells; Cetuximab 0.5ug/ml

Movie S3.

Movie of FaDu cells (stained green by calcein AM) being co-cultured with NK cells; Cetuximab 1ug/ml
